# Association of Cooking Behaviors and Kitchen Particulate Matter with Cognitive Function: A Qualitative and Quantitative Study

**DOI:** 10.3390/toxics14030227

**Published:** 2026-03-06

**Authors:** Huanxiang Zhang, Bota Baheti, Yuan Tian, Wei Liao, Yinghao Yuchi, Linlin Li, Jian Hou, Zhenxing Mao, Yuqian Li, Chongjian Wang

**Affiliations:** 1 Department of Epidemiology and Biostatistics, College of Public Health, Zhengzhou University, Zhengzhou 450001, China; zhx1979686603@126.com (H.Z.); botabht@163.com (B.B.); ty163email@163.com (Y.T.); wliaotr@163.com (W.L.); nmmqycdh@163.com (Y.Y.); lilinlin563@163.com (L.L.); 13667176505@163.com (J.H.); maozhr@gmail.com (Z.M.); 2 Department of Clinical Pharmacology, School of Pharmaceutical Science, Zhengzhou University, Zhengzhou 450001, China

**Keywords:** cooking fuel, cooking duration, kitchen ventilation, particulate matter, cognitive dysfunction

## Abstract

The effects of cooking duration and the combined effects of cooking fuel, cooking duration, and ventilation remain unclear, particularly in relation to evidence from measured kitchen particulate matter (PM) exposure. Data were sourced from the Henan Rural Cohort Study and Panel study. Cognitive function was assessed using the Mini-Mental State Examination (MMSE). Cooking fuel, cooking duration, and kitchen ventilation were obtained, and kitchen PM was monitored using U-MINI208. In qualitative analysis, 9403 participants were enrolled. Individuals with long cooking durations scored 0.36 points lower than those with short ones. Those using solid fuels, particularly with long cooking durations and poor ventilation, had the lowest cognitive scores (*β* = −2.12) and the highest cognitive dysfunction (CD) risk (*OR* = 1.88). In quantitative analysis, 135 households and 52 individuals were enrolled. Households utilizing solid fuels, longer cooking durations, or natural ventilation showed significantly increased PM concentrations, and elevated kitchen particulate levels are associated with a decline in MMSE scores. Solid fuel, long cooking duration, and poor ventilation are associated with lower cognitive function, highlighting the importance of transitioning to cleaner energy sources, reducing cooking duration, and improving kitchen environments to protect cognition.

## 1. Introduction

Cognitive dysfunction (CD) refers to impairments in cognitive domains such as memory, judgment, attention, and language, lying between normal aging and dementia. With rising life expectancy and an aging global population, age-related disorders like dementia are becoming major public health challenges [1]. In 2019, an estimated 57 million people worldwide lived with dementia, two-thirds residing in low- and middle-income countries (LMICs), and China accounted for 27% of global cases [2]. The World Health Organization (WHO) reports nearly 10 million new dementia cases annually, projecting a doubling to 153 million globally by 2050 [3]. Dementia care imposes a heavy economic burden on families and society, with global healthcare costs nearing 1 trillion USD annually, expected to double by 2030 [4,5]. As dementia cases rise, the associated economic and caregiving demands will further intensify the global burden. Given the lack of optimal treatments, identifying CD is critical for delaying dementia progression [6].

The 2024 Lancet Commission on dementia prevention, intervention, and care highlights that air pollution is one of the 14 leading modifiable risk factors for dementia [7]. Indoor air pollution is estimated to cause 3.2 million premature deaths annually [8]. Indoor air pollution, primarily caused by the combustion of solid fuels, has been identified as one of the top ten leading risk factors for global disease burden [9,10,11,12]. Incomplete combustion of solid fuels releases harmful substances like particulate matter (PM), carbon monoxide (CO), sulfur dioxide (SO_2_), nitrogen dioxide (NO_2_), and formaldehyde, contributing to indoor air pollution [13]. Solid fuels affect over 2.1 billion (around a third of the global population) people worldwide, with approximately 500 million of them residing in China [8,14].

Although studies have explored the relationship between indoor air pollution and cognitive function, the effect of cooking duration and the combined effects of cooking duration, kitchen ventilation, and fuel type on cognitive function remain unexplored [15,16,17,18]. Moreover, existing studies on fuel type transitions and usage duration remain limited. More importantly, there is currently a lack of quantitative research on indoor air pollution and cognition. Rural areas are high-risk zones for both CD and regions where solid fuels are widely used. Elderly individuals, as a high-risk group for CD, are more susceptible to the effects of solid fuels due to prolonged indoor activities.

To address this, the Henan Rural Cohort is utilized to achieve three objectives: first, to evaluate the independent and combined influences of cooking fuel, cooking time, and kitchen ventilation on cognitive function; second, to analyze the relationship between fuel conversion, its duration of use, and cognitive performance; and third, to quantitatively assess the effect of indoor PM exposure on cognitive health. The ultimate goal is to inform prevention and management strategies for CD in rural settings.

## 2. Materials and Methods

### 2.1. Study Population

As cognitive function assessment was not conducted in the first survey, the qualitative study utilized data from the second wave of the Henan Rural Cohort study (2018–2022). The first wave of this cohort recruited 39,259 adults in 2015–2017, while the second wave included 35,995 participants, of whom 28,628 completed face-to-face interviews. Further details have been published in previous literature [19,20]. The quantitative study data were derived from the first wave (completed in 2022) of a panel study established at the Zhumadian City research site within the Henan Rural Cohort, and a total of 139 households (284 participants) were recruited. After inclusion and exclusion criteria, a total of 9403 participants were included for qualitative analysis, while in quantitative analysis, 135 households were included for kitchen particulate matter concentration analysis, and 52 individuals were included for the analysis of the association between kitchen particulate matter and cognitive function scores (Figure S1).

### 2.2. Assessment of Fuel Type, Cooking Duration, and Ventilation Type

Trained investigators conducted face-to-face interviews using a standardized questionnaire to collect data on participants’ cooking fuel type, cooking duration, and kitchen ventilation. Questions covered whether participants cooked at home in the past year, the primary cooking fuel used, average cooking frequency per week, average cooking duration per meal, and the primary ventilation method in the kitchen. Cooking fuel types were classified as clean fuel (electricity, natural gas, marsh gas, or liquefied petroleum gas) and solid fuel (coal, wood, or crop residue). Daily cooking duration was categorized as short (<1.5 h/day) or long (≥1.5 h/day) based on the median daily cooking time. The daily cooking duration was calculated as the weekly cooking frequency multiplied by the cooking time of the cooking day, divided by 7. Kitchen ventilation type was classified into good (mechanical ventilation: exhaust fan/range hood) and poor (natural ventilation: window/door/chimney without mechanical ventilation). Based on cooking fuel usage in the first- and second-wave surveys, fuel transitions were categorized as clean-clean, solid-clean, clean-solid, and solid-solid. Cooking fuel usage time was classified into ≤10 years and >10 years for both clean and solid fuel.

### 2.3. Assessment of Kitchen Particulate Matter

The PM includes PM_2.5_ and PM_10_. Continuous real-time monitoring was conducted for 3 days using the U-MINI208 micro air quality monitor (Shanghai Lanju Intelligent Technology Co., Ltd., Shanghai, China). The monitoring device had a measurement interval of 1 min per data point, recording 1440 data points per day. The device data were uploaded to a cloud server (http://122.112.234.240/login, accessed on 28 February 2026) via a 4G network in real time. After each monitoring session, the data were downloaded from the backend, and the three-day average PM levels were calculated. In addition, based on the participants’ daily cooking time and the real-time monitored data, the cooking time-weighted average of kitchen PM was calculated using the following formula:

$$C_{cooking}=\frac{\sum_{i}^{n}C_{i}\times t_{i}}{\sum_{i}^{n}t_{i}}$$

*C_cooking_* is the cooking-time-weighted average of PM, *C_i_* is the average of PM during each cooking period, and *t_i_* is the corresponding cooking duration.

### 2.4. Cognitive Function Assessment

Cognitive function was assessed by the Chinese version of the Mini-Mental State Examination (MMSE), which contains five domains: orientation, memory, attention and numeracy, recall, and language. The total score is 30 points, with higher scores indicating better cognitive function. CD is defined as individuals who are illiterate with MMSE ≤ 17; individuals with a primary school education and MMSE ≤ 20; or individuals with a junior high school education or above and MMSE ≤ 24 [21,22].

### 2.5. Covariates

Covariates adjusted in this study encompass demographic information, socioeconomic status, lifestyle factors, disease history, and anthropometric [23]. Demographic information includes age, sex (men, women), and marital status (married/cohabiting, divorced/widowed/unmarried). Socioeconomic status includes education level (elementary school or below, junior school or above), and monthly income (<500, 500–999, ≥1000 RMB). Lifestyle factors comprised physical activity, which was divided into three levels (low, moderate, high) based on the International Physical Activity Questionnaire (IPAQ), smoking/drinking status (never/former, current), high-fat diet (≥75 g/day), vegetable/fruit intake (≥500 g/day), and sleep duration. Disease’s history includes psychological status (anxiety/depression), and common chronic diseases (hypertension, diabetes, dyslipidemia, stroke, and coronary heart disease). All of the above information was collected by well-trained staff using a standardized questionnaire. Anthropometric measurements included height and weight, and body mass index (BMI) was calculated as weight (kg) divided by height squared (m^2^).

### 2.6. Statistical Analyses

Continuous variables conforming to a normal distribution were expressed as mean ± standard deviation, with inter-group differences assessed using the *t*-test; otherwise, they were described as median (25th percentile, 75th percentile), and using the Wilcoxon rank-sum test. Categorical variables were presented as frequencies (percentages), and the χ^2^ test was used to compare group distributions.

In the qualitative study, a generalized linear model was used to estimate the regression coefficient (*β*) and odds ratio (*OR*) of cooking fuel with cognitive function. Subgroup analysis was conducted to explore whether sociodemographic characteristics, socioeconomic status, lifestyle factors, and disease status have a modifying effect on the association between cooking fuel and cognitive function, and *p*-values for the multiplicative interaction of each stratified variable and cooking fuel on cognitive function were calculated. Two sensitivity analyses were conducted to test robustness. First, to address potential selection bias due to missing data, multiple imputations (25 imputations) were used to reduce bias and improve accuracy. Missing values for covariates were replaced with imputed estimates, and results from imputed datasets were combined. Second, to exclude the potential impact of brain injury on the association between cooking fuel and cognitive function, stroke patients were excluded from the sensitivity analysis.

In a quantitative study, the cognitive score conforms to the normal distribution, while the exposure to kitchen PM does not (Table S1). Therefore, Spearman’s rank correlation was used to analyze the association between kitchen PM exposure and cognitive scores. Generalized linear regression was employed to estimate the *β* of kitchen PM on cognitive scores.

All statistical analyses were performed using R software version 4.3.2, with a significance level of α = 0.05 (two-sided test).

## 3. Results

### 3.1. Qualitative Study

#### 3.1.1. Basic Characteristics of Participants

Table 1 presents characteristics of 9403 participants, including 2996 with CD. Those with CD were more likely to be female, illiterate, have a low income, high fat intake, low fruit and vegetable intake, lower BMI, longer sleep, experience anxiety/depression, and have multiple chronic diseases (*p* < 0.05); they also tended to use solid cooking fuels and rely on natural ventilation (*p* < 0.05). In contrast, being married and engaging in moderate physical activity were associated with better cognitive function (*p* < 0.05). Solid fuel users had lower cognitive scores across various domains and a higher prevalence of cognitive dysfunction compared to clean fuel users (*p* < 0.001), as shown in Table S2.

#### 3.1.2. Independent Association of Cooking Fuel, Daily Cooking Duration, and Kitchen Ventilation with Cognitive Function

**Figure 1:**
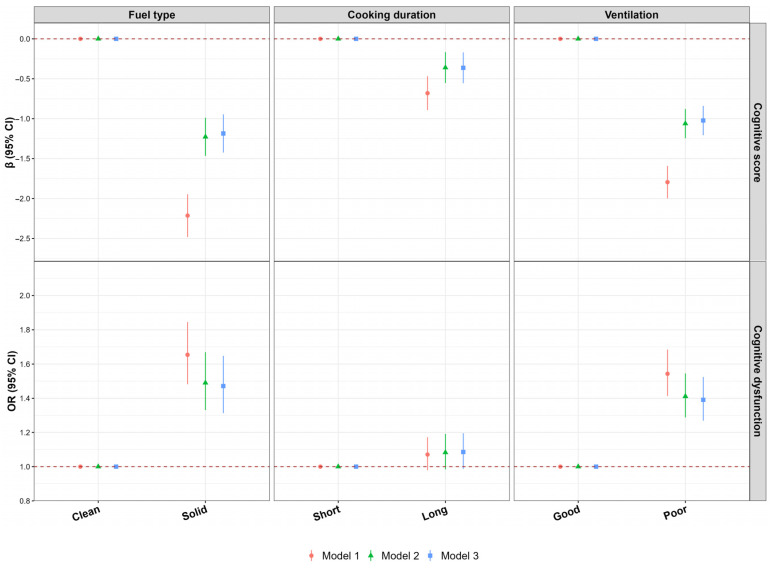
Independent association of cooking fuel, daily cooking duration, and kitchen ventilation with cognitive function. Short: cooking duration < 1.5 h/day; Long: cooking duration ≥ 1.5 h/day; Good: mechanical ventilation; Poor: natural ventilation. Abbreviations: *β*, regression coefficient; *OR*, odds ratio; *CI*, confidence interval. Model 1 was unadjusted. Model 2 was adjusted for age, sex, marital status, education level, per capita monthly household income, smoking status, drinking status, physical activity level, high-fat diet, higher intake of fruits and vegetables, body mass index, and night sleep duration. Model 3 was further adjusted for anxiety/depression and the number of chronic diseases.

Independent association of cooking fuel, daily cooking duration, and kitchen ventilation with cognitive function was shown in Figure 1. The use of solid cooking fuels, daily cooking time ≥ 1.5 h/day, and natural ventilation are statistically significantly associated with lower cognitive scores, with solid cooking fuels and natural ventilation also showing a significant positive correlation with CD, while the positive correlation between daily cooking time ≥ 1.5 h/day and CD is not statistically significant. In the fully adjusted model, the cognitive score of solid fuel users is 1.18 lower and the CD risk is 0.47 times higher than fuel users (Figure 1 and Table S3); participants with cooking duration ≥ 1.5 h/day had a cognitive score 0.36 lower than those cooking duration < 1.5 h/day (Figure 1 and Table S4); the cognitive score of mechanical ventilation users is 1.02 lower and the CD risk is 0.39 times higher than natural ventilation users (Figure 1 and Table S5). Solid fuel use was generally linked to lower cognitive scores and a higher risk of CD in most subgroups, as shown in Figure S2. Two sensitivity analyses showed that the association between solid fuel and cognitive function remains unchanged (Tables S6 and S7), and solid fuel was associated with lower orientation, attention, numeracy, recall, and language scores (Table S8). Notably, the combined exposure to these three factors exerts a stronger association with cognitive function decline compared to single-factor exposure, as detailed in the following combined effect analysis.

#### 3.1.3. Combined Association of Cooking Fuel, Daily Cooking Duration, and Kitchen Ventilation with Cognitive Function

**Figure 2:**
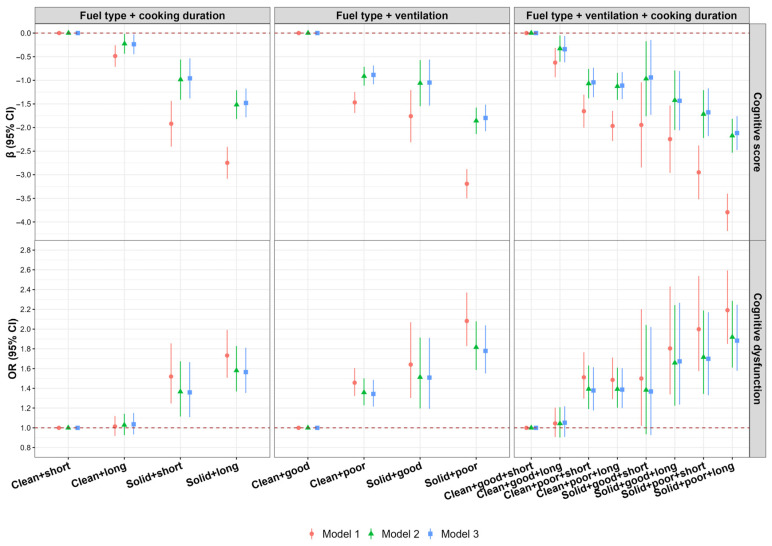
Combined associations of cooking fuel type, daily cooking duration, and kitchen ventilation with cognitive function. Short: cooking duration < 1.5 h/day; Long: cooking duration ≥ 1.5 h/day; Good: mechanical ventilation; Poor: natural ventilation. Abbreviations: *β*, regression coefficient; *OR*, odds ratio; *CI*, confidence interval. Model 1 was unadjusted. Model 2 was adjusted for age, sex, marital status, education level, per capita monthly household income, smoking status, drinking status, physical activity level, high-fat diet, higher intake of fruits and vegetables, body mass index, and night sleep duration. Model 3 was further adjusted for anxiety/depression and the number of chronic diseases.

The combined effect of cooking fuel, cooking duration, and kitchen ventilation on cognitive function were depictured in Figure 2. Compared to clean fuel users with short cooking duration, solid fuel users with long cooking duration had the most significant decline in cognitive scores (*β* = −1.48, 95% *CI*: −1.78, −1.18) and the highest CD risk (*OR* = 1.56, 95% *CI*: 1.35, 1.81), as shown in Figure 2 and Table S9. Compared to clean fuel users and natural ventilation, solid fuel users with poor ventilation showed the largest decline in cognitive scores (*β* = −1.80, 95% *CI*: −2.08, −1.51), and the highest CD risk (*OR* = 1.78, 95% *CI*: 1.55, 2.04), as shown in Figure 2 and Table S10. Compared to clean fuel users with good ventilation and short cooking duration, the cognitive scores of the remaining seven groups decreased significantly, and the risk of CD was significantly increased in the remaining 5 groups (except for clean + good + long group and solid + good + short group), with participants using solid fuel, poor ventilation, and short cooking duration had the most significant decline in cognitive scores (*β* = −2.12, 95% *CI*: −2.48, −1.76) and the highest CD risk (*OR* = 1.88, 95% *CI*: 1.58, 2.25), as shown in Figure 2 and Table S11).

#### 3.1.4. Association of Cooking Fuel Transition and Cooking Fuel Usage Time with Cognitive Function

After adjusting for various confounding factors, compared to clean-clean cooking fuel users, those who use solid-clean, clean-solid, or solid-solid cooking fuel exhibited significantly lower cognitive scores and a significantly increased risk of CD, as shown in Figure S3. Among them, individuals who switched from clean fuels to solid fuel and consistently used solid fuel showed more pronounced declines in cognitive scores, with *β* and 95% (*CI*) of −0.77 (−1.36, 0.17) and −1.36 (−1.81, −0.92), respectively, and higher risks of CD, with *OR* and 95% (*CI*) of 1.53 (1.16, 2.03) and 1.63 (1.32, 2.01), respectively.

After adjusting for various confounding factors (Model 3), individuals using clean cooking fuel for ≤ 10 years, those using clean fuel for > 10 years, solid fuel for ≤ 10 years, and solid fuel for > 10 years exhibited significantly lower cognitive scores and a higher risk of CD, as shown in Figure S4. Among them, individuals who have been using solid fuel for ≤ 10 years and those using solid fuel for > 10 years showed more pronounced declines in cognitive scores, with *β* and 95% (*CI*) of −1.29 (−1.76, −0.82) and −1.23 (−1.50, −0.96), respectively, and higher risks of cognitive dysfunction, with *OR* and 95% (*CI*) of 1.44 (1.15, 1.80) and 1.50 (1.32, 1.71), respectively.

### 3.2. Quantitative Study

#### 3.2.1. Distribution of Kitchen Particulate Matter Concentrations

**Figure 3:**
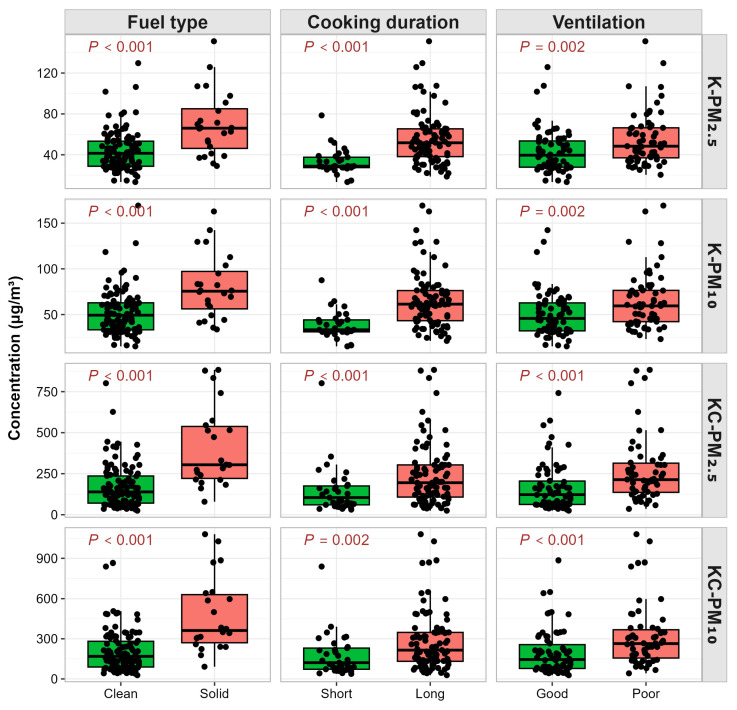
Comparison of kitchen particulate matter concentrations among different cooking fuels, cooking durations, and ventilation types. Short: cooking duration < 1.5 h/day; Long: cooking duration ≥ 1.5 h/day; Good: mechanical ventilation; Poor: natural ventilation. Abbreviations: K-PM_2.5_, daily average kitchen PM_2.5_; K-PM_10_, daily average kitchen PM_10_; KC-PM_2.5_, cooking-time-weighted kitchen PM_2.5_; KC-PM_10_, cooking-time-weighted kitchen PM_10_.

This study monitored the kitchen daily average of PM in 135 households. Median for daily average of PM_2.5_ and PM_10_ was 43.71 μg/m^3^ and 51.21 μg/m^3^, respectively. For the 128 households with complete cooking-time PM, the median for the cooking-time-weighted average was substantially higher: 162.69 μg/m^3^ for PM_2.5_ and 196.16 μg/m^3^ for PM_10_ (Table S12). Daily average of PM_2.5_ and PM_10_ were significantly higher in households using solid fuels (median: 66.02 μg/m^3^ vs. 41.45 μg/m^3^ forPM_2.5_, and 75.54 μg/m^3^ vs. 49.28 μg/m^3^ forPM_10_), cooking for ≥1.5 h/day (median:51.73 μg/m^3^ vs. 28.86 μg/m^3^ forPM_2.5_, and 61.40 μg/m^3^ vs. 33.32 μg/m^3^ forPM_10_), or relying on natural ventilation (median:48.40 μg/m^3^ vs. 39.58 μg/m^3^ forPM_2.5_, and 59.47 μg/m^3^ vs. 45.79 μg/m^3^ forPM_10_) compared to those using clean fuels, cooking for <1.5 h/day, or using mechanical ventilation (*p* ≤ 0.002; Figure 3 and Table S13). Similar patterns were observed for cooking-time-weighted average (Figure 3 and Table S14).

#### 3.2.2. Association of Kitchen Particulate Matter with Cognitive Function

**Figure 4:**
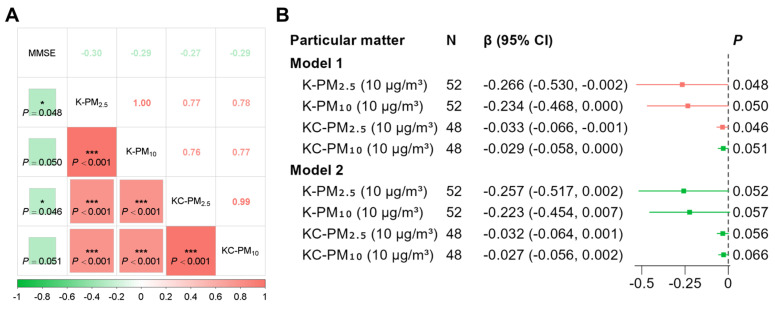
Association of kitchen particulate matter and cognitive function scores. (**A**) Spearman correlation analysis between cognitive function scores and kitchen particulate matter (*: *p* < 0.05; ***: *p* < 0.001). (**B**) General linear regression analysis between cognitive function scores and kitchen particulate matter. Abbreviations: K-PM_2.5_, daily average kitchen PM_2.5_; K-PM_10_, daily average kitchen PM_10_; KC-PM_2.5_, cooking-time-weighted kitchen PM_2.5_; KC-PM_10_, cooking-time-weighted kitchen PM_10_; *β*, regression coefficient; *CI*, confidence interval. Model 1 was unadjusted. Model 2 was adjusted for age and sex.

A total of 52 individuals were included, including 13 men and 39 women. The average age and MMSE scores were 68.62 years and 24.96 points, the median for the daily average of PM_2.5_ and PM_10_ was 45.62 μg/m^3^ and 55.44 μg/m^3^, and the cooking-time-weighted average was 231.43 μg/m^3^ and 272.52 μg/m^3^ (Table S15). MMSE score was significantly negatively correlated with the daily average of PM_2.5_ and PM_10_, while the cooking-time-weighted average of PM_2.5_ (correlation coefficient (r) was −0.27, −0.28, and −0.28; a marginally significant negative correlation was observed with the cooking-time-weighted average of PM_2.5_ (r = −0.28), as shown in Figure 4A. In the crude model, the daily average of PM_2.5_ and PM_10_ and the cooking-time-weighted average of PM_2.5_ were significantly negatively associated with MMSE score (*β* was 0.266, 0.234, and 0.33, respectively), while the cooking-time-weighted average of PM_10_ showed a marginal association (Figure 4B). These associations generally remained with marginal significance even after adjusting for age and sex. In order to reduce sample loss, we analyzed 140 participants who underwent MMSE examinations. The results showed that kitchen PM was negatively correlated with the MMSE score, but this correlation was statistically significant and marginally statistically significant only in terms of the daily average of PM_2.5_ and PM_10_ (Figure S5).

## 4. Discussion

This study reveals for the first time that prolonged daily cooking duration is associated with lower cognitive scores, and that this association between using solid fuel and cognition is most pronounced among individuals with both long cooking durations and natural kitchen ventilation. Furthermore, persistent solid fuel use exerts the greatest negative impact on cognitive function, and those using solid cooking fuels for > 10 years face the highest *OR* for CD. The research also provides the first evidence that households using solid fuel, cooking for extended periods, or relying on natural ventilation have significantly higher kitchen PM concentrations, which are significantly linked to declines in MMSE scores.

Lower cognitive scores across various domains in adult solid fuel users were first reported in a 2018 study from MHAS [15]. Subsequent studies from India, China, Mexico, and Ireland have also found an association between solid cooking fuel use and poorer cognitive function [24,25,26]. Multi-cohort studies have similarly reached consistent conclusions [27]. A representative cohort study from China also found that using solid fuel for cooking is independently associated with an increased risk of dementia [28]. In contrast, the current study focuses on elderly rural residents, a high-risk population in regions with a high prevalence of CD and solid cooking fuel use, providing epidemiological evidence for the association between indoor air pollution from solid cooking fuel use and cognitive function, and offering scientific support for the prevention and management of CD in rural populations. Furthermore, this study found that participants who relied on poor ventilation was associated with a higher risk of CD, which is consistent with the CLHLS, LASI, and SAGE studies [27,29,30,31,32]. Another study showed that air pollution levels in poorly ventilated kitchens were 20 times higher than in well-ventilated ones [33].

A novel finding of this study is that prolonged cooking duration is associated with lower cognitive scores. Although no direct research has explored the association between cooking duration and cognitive function, studies suggest that long cooking duration is linked to adverse health outcomes and raised kitchen PM_2.5_ levels [34,35,36]. Our previous study also shows that long cooking duration increases the risk of sleep disorders and anxiety/depression [37]. The degree of association between different cooking-related factors and cognitive scores varied (*β* range: −0.36 to −1.18), reflecting the heterogeneity of these factors. Solid fuel showed the strongest association, consistent with its high pollutant emissions, while cooking duration was more modest and likely dependent on fuel type and ventilation conditions. Another novel finding is that individuals characterized by the combination of solid fuel use, natural ventilation, and a cooking duration of ≥1.5 h/day exhibited the highest risk of CD. This suggests that comprehensive cooking behavior interventions may be more beneficial than single-factor approaches. Although no previous study has explored the combined effects of these three cooking behaviors, existing research has demonstrated that the association between solid fuel use and adverse health outcomes is more pronounced among individuals with poor ventilation or those with a cooking duration of ≥1.5 h/day [38,39,40]. These studies provide evidence that long cooking duration and poor kitchen ventilation may exacerbate the negative association between solid cooking fuel and cognitive function.

Cognitive function is worst among consistently solid fuel users, followed by those who switched from clean to solid users, which was similar to CHARLS, CHNS, and CLHLS [29,41,42]. Only one small sample size study evaluated the relationship between cooking fuel duration and cognitive function, finding that current and long-term intensities of indoor solid fuel use were associated with lower overall and most domain-specific cognitive outcomes [43]. Our study found that compared to those who used clean fuels for ≤10 years, individuals who used solid fuels for >10 years faced a higher CD risk. For each additional year of using polluting fuels, the risk of decline in intrinsic abilities increases by 1.243 times, with the follow-up interval used as the duration of fuel use [44]. These studies suggest that solid fuels may have a cumulative effect on CD, but considering that CD is an age-related disease, although age was adjusted for, more rigorously designed cohort studies are still needed to explore the cumulative effects of solid cooking fuels on cognition.

One pioneering finding was that higher kitchen PM is associated with lower MMSE scores, providing more concrete evidence of the direct cognitive impact of indoor air pollution and supporting cooking-related particulate exposure as a significant environmental factor in cognitive decline. We also found that households using solid fuels, long cooking duration, or relying on natural ventilation had higher PM concentrations, which was also observed in India [33]. Recent studies have found that fuel type is associated with indoor PM_2.5_ and personal PM_2.5_ exposure, and increased personal PM_2.5_ exposure and the household use of polluting cooking fuels are associated with lower scores in children’s KABC-II neurocognitive assessment and VABS-3 adaptive behavior assessment [45]. However, it is important to acknowledge that another study found no association between household air pollution exposure and neurodevelopmental outcomes [46]. To our knowledge, our study is the first to specifically explore the association between accurately monitored kitchen PM_2.5_ pollution and cognitive function in the elderly.

Although the specific mechanisms underlying the association between solid fuel and cognitive function remain unclear, several hypotheses have been proposed. The pollutants released during the incomplete combustion of solid fuels, such as PM, CO, and NO_2_, may enter the body via the respiratory tract, potentially influencing brain processes like Aβ protein aggregation and tau protein hyperphosphorylation, which could lead to cognitive impairment [47]. Long-term exposure to air pollutants may also alter brain morphology, causing damage to both white and gray matter [48,49]. Additionally, air pollution could activate microglia in the central nervous system, triggering systemic inflammation, oxidative stress, and DNA oxidative damage, which release inflammatory cytokines and contribute to neuroinflammation, degenerative changes, and blood–brain barrier dysfunction, ultimately affecting cognitive function [50,51,52]. The discussed biological pathways represent reasonable inferences based on current evidence and require further validation through additional basic research and epidemiological investigations.

This study also had several limitations. First, the cross-sectional design limits causal inferences between solid fuel exposure and cognitive function, and future prospective studies are needed. Second, cognitive function was assessed using the MMSE rather than more precise neuroimaging techniques such as computed tomography. Although the MMSE has limitations, it has high acceptance and good validity (87% sensitivity, 82% specificity) and is widely used in large-scale epidemiological surveys [53]. Third, although the assessment of cooking behaviors relied primarily on self-report, which may introduce recall bias and social desirability bias, we conducted in-home observations to verify certain information and thus help minimize potential recall errors. Fourth, this study did not capture the specific timing of fuel type transitions, limiting the assessment of their impact. Fifth, the pollutants released during cooking form a complex mixture of PM and various gaseous hazardous substances. Therefore, using only PM as an exposure indicator may underestimate the total impact of kitchen air pollution on cognitive function. Sixth, kitchen PM concentration was measured over three consecutive days, a feasible but limited window that may not capture long-term habitual exposure or account for seasonal and fuel-use fluctuations. Future studies should aim for extended monitoring periods to better characterize long-term exposure. Finally, the small sample size for measuring kitchen PM exposure may limit the statistical power and generalizability of the analytical results; therefore, these analyses should be considered exploratory. The findings provide preliminary evidence regarding potential underlying pathways, but warrant confirmation in future studies with larger samples and more comprehensive exposure assessment.

## 5. Conclusions

This study provides strong epidemiological evidence linking cooking-related exposures to cognitive health among rural elderly individuals. It demonstrates that the use of solid fuels, prolonged daily cooking duration, and reliance on natural ventilation are independently associated with lower cognitive scores and an increased risk of CD, with the most pronounced cognitive decline observed in individuals exposed to all three risk factors simultaneously. Sustained use of or switching to solid fuels further aggravates cognitive deterioration, suggesting a potential cumulative effect. Preliminary quantitative analysis confirms that higher concentrations of PM_2.5_ and PM_10_ in kitchens are negatively correlated with MMSE scores, thereby providing mechanistic support for the role of cooking-related indoor pollution as an environmental mediator of cognitive decline. Although acknowledging the limitations inherent in cross-sectional design, the findings highlight that reducing kitchen air pollution represents a viable public health approach to preserving cognitive function in the aging rural population.

## Figures and Tables

**Table 1:** Basic characteristics of normal cognition and cognitive dysfunction in a qualitative study.

Characteristics	Total (n = 9403)	Normal Cognition (n = 6407)	Cognitive Dysfunction (n = 2996)	*p*
Age (year), mean ± SD	68.56 ± 5.27	68.23 ± 5.02	69.25 ± 5.71	<0.001
Women, n (%)	7198 (76.55)	4830 (75.39)	2368 (79.04)	<0.001
Married/Cohabitation, n (%)	7274 (77.36)	5036 (78.60)	2238 (74.70)	<0.001
Elementary school or above, n (%)	6124 (65.13)	4249 (66.32)	1875 (62.58)	<0.001
Average monthly income (RMB), n (%)				<0.001
<500	3961 (42.12)	2548 (39.77)	1413 (47.16)	
500~	2545 (27.07)	1760 (27.47)	785 (26.20)	
1000~	2897 (30.81)	2099 (32.76)	798 (26.64)	
Current smoker, n (%)	855 (9.09)	622 (9.71)	233 (7.78)	0.003
Current drinker, n (%)	705 (7.50)	510 (7.96)	195 (6.51)	0.014
Physical activity, n (%)				<0.001
Low	3552 (37.78)	2330 (36.37)	1222 (40.79)	
Moderate	3975 (42.27)	2802 (43.73)	1173 (39.15)	
High	1876 (19.95)	1275 (19.90)	601 (20.06)	
High fat diet, n (%)	1354 (14.40)	976 (15.23)	378 (12.62)	0.0001
High vegetable and fruit intake, n (%)	4109 (43.70)	2888 (45.08)	1221 (40.75)	<0.001
BMI (kg/m^2^), mean ± SD	24.60 ± 3.58	24.70 ± 3.58	24.37 ± 3.57	<0.001
Night sleep duration (h/day), mean ± SD	7.62 ± 1.35	7.54 ± 1.28	7.78 ± 1.47	<0.001
Anxiety/depression, n (%)	559 (5.94)	331 (5.17)	228 (7.61)	<0.001
Number of chronic diseases, n (%)				<0.001
0	2722 (28.95)	1890 (29.50)	832 (27.77)	
1	3301 (35.11)	2288 (35.71)	1013 (33.81)	
2	2157 (22.94)	1475 (23.02)	682 (22.76)	
3~	1223 (13.01)	754 (11.77)	469 (15.65)	
Solid fuel, n (%)	1646 (17.51)	966 (15.08)	680 (22.70)	<0.001
Natural ventilation, n (%)	4922 (52.34)	3135 (48.93)	1787 (59.65)	<0.001
Cooking duration ≥ 1.5 h/day, n (%)	5822 (61.92)	3934 (61.40)	1888 (63.02)	0.133

Abbreviations: SD, standard deviation; RMB, Renminbi; BMI, body mass index. A *t*-test was performed to compare the differences in continuous variables; a chi-square test was used to compare the differences in the categorical variables.

## Data Availability

The original contributions presented in this study are included in the article/Supplementary Material. Further inquiries can be directed to the corresponding authors.

## Supplementary Materials

The following supporting information can be downloaded at: https://www.mdpi.com/article/10.3390/toxics14030227/s1. Table S1: shapiro–Wilk test for normality on cognitive scores and kitchen particulate matter in quantitative analysis. Table S2: cognitive function in different cooking fuel groups; Table S3: independent association of cooking fuel with cognitive function; Table S4: independent association of daily cooking duration with cognitive function; Table S5. independent association of kitchen ventilation with cognitive function; Table S6: association of cooking fuel with cognitive function after imputing missing covariates; Table S7: association of cooking fuel with cognitive function after excluding stroke participants; Table S8: association of cooking fuel with cognitive score in different domains; Table S9: combined association of cooking fuel and cooking duration with cognitive function; Table S10: combined association of cooking fuel and kitchen ventilation with cognitive function; Table S11: combined association of cooking fuel, daily cooking duration and kitchen ventilation with cognitive function; Table S12: the distribution of particulate matter concentration in the kitchen; Table S13: comparison of kitchen particulate matter concentrations among different cooking fuels, cooking durations, and ventilation types; Table S14: comparison of cooking-time-weighted kitchen particulate matter concentrations among different cooking fuels, cooking durations, and ventilation types; Table S15: the basic characteristics of the subjects included in the quantitative analysis; Figure S1: flow chart of study participants ((A): flow chart of qualitative study participants;(B): flow chart of quantitative study participants); Figure S2: stratification analyses of association between cooking fuel and cognitive function; Figure S3: association of cooking fuel transition with cognitive function; Figure S4: association of cooking fuel usage time with cognitive function; Figure S5: association of kitchen particular matter and cognitive function scores.
