# Supplementary material for: Association of Cooking Behaviors and Kitchen Particulate Matter with Cognitive Function: A Qualitative and Quantitative Study
Source: Toxics. 2026 Mar 6;14(3):227. doi: 10.3390/toxics14030227 (PMC13030482; doi:10.3390/toxics14030227)
Supplement: Supplementary file 1 [file toxics-14-00227-s001.zip › toxics-4153216-supplementary.pdf]

Supplemental Materials

Legend

Table S1. Shapiro–Wilk test for normality on cognitive scores and kitchen particulate matter in quantitative analysis.

Table S2. Cognitive function in different cooking fuel groups.

Table S3. Independent association of cooking fuel with cognitive function.

Table S4. Independent association of daily cooking duration with cognitive function.

Table S5. Independent association of kitchen ventilation with cognitive function.

Table S6. Association of cooking fuel with cognitive function after imputing missing covariates.

Table S7. Association of cooking fuel with cognitive function after excluding stroke participants.

Table S8. Association of cooking fuel with cognitive score in different domains.

Table S9. Combined association of cooking fuel and cooking duration with cognitive function.

Table S10. Combined association of cooking fuel and kitchen ventilation with cognitive function.

Table S11. Combined association of cooking fuel, daily cooking duration, and kitchen ventilation with cognitive function.

Table S12. The distribution of particulate matter concentration in the kitchen.

Table S13. Comparison of kitchen particulate matter concentrations among different cooking methods, fuels, cooking durations, and ventilation types.

Table S14. Comparison of cooking-time-weighted kitchen particulate matter concentrations among different cooking fuels, cooking durations, and ventilation types.

Table S15. The basic characteristics of the subjects included in the quantitative analysis.

Figure S1. Flow chart of study participants.

Figure S2. Stratification analyses of the association between cooking fuel and cognitive function.

Figure S3. Association of cooking fuel transition with cognitive function.

Figure S4. Association of cooking fuel usage time with cognitive function.

Figure S5. Association of kitchen particulate matter and cognitive function scores.

Table S1. Shapiro-Wilk test for normality on cognitive scores and kitchen particulate matter in quantitative analysis.

Variables	N	Statistic	P
Cognitive scores	52	0.963	0.11
Daily average kitchen $PM_{2.5}$	52	0.834	0
Daily average kitchen PM_{10}	52	0.86	0
Cooking-time-weighted kitchen $PM_{2.5}$	48	0.823	0
Cooking-time-weighted kitchen PM_{10}	48	0.837	0

Table S2. Cognitive function in different cooking fuel groups.

Characteristics	Total (n =9,403)	Clean fuel (n = 7,757)	Solid fuel (n = 1,646)	*P*
Cognitive score in different domains				
Orientation, mean ± SD	8.29 ± 2.16	8.44 ± 2.07	7.59 ± 2.44	< 0.001
Memory, mean ± SD	2.74 ± 0.73	2.76 ± 0.71	2.66 ± 0.84	< 0.001
Attention and numeracy, mean ± SD	2.51 ± 1.98	2.59 ± 1.97	2.09 ± 1.93	< 0.001
Recall ability, mean ± SD	1.51 ± 1.21	1.56 ± 1.20	1.29 ± 1.20	< 0.001
Verbal ability, mean ± SD	7.39 ± 1.24	7.48 ± 1.20	6.98 ± 1.31	< 0.001
Total cognitive score, mean ± SD	22.44 ± 5.10	22.83 ± 4.94	20.62 ± 5.42	< 0.001
Cognitive dysfunction, n (%)	2996 (31.86)	2316 (29.86)	680 (41.31)	< 0.001

Abbreviations: SD, standard deviation.

Continuous variables were represented by the mean ± standard deviation, and the t-test was used to compare differences. Categorical variables were represented by the number of participants (percentage), and the chi-square test was used to compare differences.

Table S3. Independent association of cooking fuel with cognitive function.

Cooking fuel type	Cognitive score		Cognitive dysfunction	
	β (95% *CI*)	*P*	*OR* (95% *CI*)	*P*
**Model 1**				
Clean fuel	Ref.		Ref.	
Solid fuel	-2.21 (-2.48, -1.95)	< 0.001	1.65 (1.48, 1.84)	< 0.001
**Model 2**				
Clean fuel	Ref.			
Solid fuel	-1.23 (-1.47, -0.99)	< 0.001	Ref.	
**Model 3**			1.49 (1.33, 1.67)	< 0.001
Clean fuel	Ref.		Ref.	
Solid fuel	-1.18 (-1.42, -0.95)	< 0.001	1.47 (1.31, 1.65)	< 0.001

Abbreviations: *β*, regression coefficient; *OR*, odds ratio; *CI*, confidence interval.

Model 1 was unadjusted.

Model 2 was adjusted for age, sex, marital status, education level, per capita monthly household income, smoking status, drinking status, physical activity level, high-fat diet, higher intake of fruits and vegetables, body mass index, and night sleep duration.

Model 3 was further adjusted for anxiety/depression, number of chronic diseases.

Table S4. Independent association of daily cooking duration with cognitive function.

Daily cooking duration	Cognitive score		Cognitive dysfunction	
	β (95% *CI*)	*P*	*OR* (95% *CI*)	*P*
**Model 1**				
Short	Ref.		Ref.	
Long	-0.68 (-0.89, -0.47)	< 0.001	1.07 (0.98, 1.17)	0.133
**Model 2**				
Short	Ref.		Ref.	
Long	-0.36 (-0.55, -0.17)	< 0.001	1.08 (0.98, 1.19)	0.103
**Model 3**				
Short	Ref.		Ref.	
Long	-0.36 (-0.56, -0.17)	< 0.001	1.09 (0.99, 1.20)	0.091

Short: cooking duration < 1.5 hours /day; Long: cooking duration ≥ 1.5 hours/day.

Abbreviations: *β*, regression coefficient; *OR*, odds ratio; *CI*, confidence interval.

Model 1 was unadjusted.

Model 2 was adjusted for age, sex, marital status, education level, per capita monthly household income, smoking status, drinking status, physical activity level, high-fat diet, higher intake of fruits and vegetables, body mass index, and night sleep duration.

Model 3 was further adjusted for anxiety/depression, number of chronic diseases.

Table S5. Independent association of kitchen ventilation with cognitive function.

Kitchen ventilation type	Cognitive score		Cognitive dysfunction	
	β (95% *CI*)	*P*	*OR* (95% *CI*)	*P*
**Model 1**				
Good	Ref.		Ref.	
Poor	-1.79 (-2.00, -1.59)	< 0.001	1.54 (1.41, 1.68)	< 0.001
**Model 2**				
Good	Ref.		Ref.	
Poor	-1.06 (-1.24, -0.88)	< 0.001	1.41 (1.29, 1.54)	< 0.001
**Model 3**				
Good	Ref.		Ref.	
Poor	-1.02 (-1.21, -0.84)	< 0.001	1.39 (1.27, 1.52)	< 0.001

Good: mechanical ventilation; Poor: natural ventilation.

Abbreviations: *β*, regression coefficient; *OR*, odds ratio; *CI*, confidence interval.

Model 1 was unadjusted.

Model 2 was adjusted for age, sex, marital status, education level, per capita monthly household income, smoking status, drinking status, physical activity level, high-fat diet, higher intake of fruits and vegetables, body mass index, and night sleep duration.

Model 3 was further adjusted for anxiety/depression, number of chronic diseases.

Table S6. Association of cooking fuel with cognitive function after imputing missing covariates.

Cooking fuel type	Cognitive score		Cognitive dysfunction	
	β (95% *CI*)	*P*	*OR* (95% *CI*)	*P*
**Model 1**				
Clean fuel	Ref.		Ref.	
Solid fuel	-2.28 (-2.54, -2.02)	< 0.001	1.66 (1.49, 1.84)	< 0.001
**Model 2**				
Clean fuel	Ref.		Ref.	
Solid fuel	-1.28 (-1.52, -1.05)	< 0.001	1.49 (1.34, 1.66)	< 0.001
**Model 3**				
Clean fuel	Ref.		Ref.	
Solid fuel	-1.24 (-1.47, -1.01)	< 0.001	1.47 (1.32, 1.64)	< 0.001

Abbreviations: *β*, regression coefficient; *OR*, odds ratio; *CI*, confidence interval.

Model 1 was unadjusted.

Model 2 was adjusted for age, sex, marital status, education level, per capita monthly household income, smoking status, drinking status, physical activity level, high-fat diet, higher intake of fruits and vegetables, body mass index, and night sleep duration.

Model 3 was further adjusted for anxiety/depression, number of chronic diseases.

Table S7. Association of cooking fuel with cognitive function after excluding stroke participants.

Cooking fuel type	Cognitive score		Cognitive dysfunction	
	β (95% *CI*)	*P*	*OR* (95% *CI*)	*P*
**Model 1**				
Clean fuel	Ref.		Ref.	
Solid fuel	-2.27 (-2.56, -1.98)	< 0.001	1.70 (1.51, 1.91)	< 0.001
**Model 2**				
Clean fuel	Ref.		Ref.	
Solid fuel	-1.25 (-1.50, -0.99)	< 0.001	1.50 (1.32, 1.70)	< 0.001
**Model 3**				
Clean fuel	Ref.		Ref.	
Solid fuel	-1.21 (-1.47, -0.95)	< 0.001	1.48 (1.30, 1.67)	< 0.001

Abbreviations: *β*, regression coefficient; *OR*, odds ratio; *CI*, confidence interval.

Model 1 was unadjusted.

Model 2 was adjusted for age, sex, marital status, education level, per capita monthly household income, smoking status, drinking status, physical activity level, high-fat diet, higher intake of fruits and vegetables, body mass index, and night sleep duration.

Model 3 was further adjusted for anxiety/depression, number of chronic diseases.

Table S8. Association of cooking fuel with cognitive score in different domains.

Cognitive score in different domains	Model 1		Model 2		Model 3	
	β (95% *CI*)	*P*	*β* (95% *CI*)	*P*	*β* (95% *CI*)	*P*
**Orientation**						
Clean fuel	Ref.		Ref.		Ref.	
Solid fuel	-0.85 (-0.96, -0.73)	< 0.001	-0.50 (-0.61, -0.40)	< 0.001	-0.49 (-0.60, -0.38)	< 0.001
**Memory**						
Clean fuel	Ref.		Ref.		Ref.	
Solid fuel	-0.09 (-0.13, -0.06)	< 0.001	-0.03 (-0.07, 0.01)	0.133	-0.03 (-0.07, 0.01)	0.158
**Attention and numeracy**						
Clean fuel	Ref.		Ref.		Ref.	
Solid fuel	-0.50 (-0.61, -0.40)	< 0.001	-0.18 (-0.28, -0.08)	< 0.001	-0.16 (-0.26, -0.07)	0.001
**Recall**						
Clean fuel	Ref.		Ref.		Ref.	
Solid fuel	-0.27 (-0.33, -0.20)	< 0.001	-0.16 (-0.22, -0.09)	< 0.001	-0.15 (-0.21, -0.08)	< 0.001
**Language**						
Clean fuel	Ref.		Ref.		Ref.	
Solid fuel	-0.50 (-0.57, -0.44)	< 0.001	-0.36 (-0.42, -0.30)	< 0.001	-0.35 (-0.42, -0.29)	< 0.001

Abbreviations: *β*, regression coefficient; *OR*, odds ratio; *CI*, confidence interval.

Model 1 was unadjusted.

Model 2 was adjusted for age, sex, marital status, education level, per capita monthly household income, smoking status, drinking status, physical activity level, high-fat diet, higher intake of fruits and vegetables, body mass index, and night sleep duration.

Model 3 was further adjusted for anxiety/depression, number of chronic diseases.

Table S9. Combined association of cooking fuel and daily cooking duration with cognitive function.

	Cognitive score		Cognitive dysfunction	
	β (95% *CI*)	*P*	*OR* (95% *CI*)	*P*
**Model 1**				
Clean + short	Ref.			
Clean + long	-0.49 (-0.71, -0.26)	< 0.001	1.01 (0.92, 1.12)	0.778
Solid + short	-1.92 (-2.40, -1.44)	< 0.001	1.52 (1.25, 1.85)	< 0.001
Solid + long	-2.75 (-3.08, -2.41)	< 0.001	1.73 (1.51, 1.99)	< 0.001
**Model 2**				
Clean + short	Ref.			
Clean + long	-0.23 (-0.44, -0.02)	0.034	1.03 (0.93, 1.14)	0.596
Solid + short	-0.98 (-1.41, -0.56)	< 0.001	1.37 (1.11, 1.67)	0.003
Solid + long	-1.52 (-1.82, -1.21)	< 0.001	1.58 (1.37, 1.83)	< 0.001
**Model 3**				
Clean + short	Ref.			
Clean + long	-0.24 (-0.45, -0.03)	0.026	1.04 (0.93, 1.15)	0.509
Solid + short	-0.96 (-1.38, -0.54)	< 0.001	1.36 (1.11, 1.67)	0.003
Solid + long	-1.48 (-1.78, -1.18)	< 0.001	1.56 (1.35, 1.81)	< 0.001

Short: cooking duration < 1.5 hours /day; Long: cooking duration ≥ 1.5 hours/day.

Abbreviations: *β*, regression coefficient; *OR*, odds ratio; *CI*, confidence interval.

Model 1 was unadjusted.

Model 2 was adjusted for age, sex, marital status, education level, per capita monthly household income, smoking status, drinking status, physical activity level, high-fat diet, higher intake of fruits and vegetables, body mass index, and night sleep duration.

Model 3 was further adjusted for anxiety/depression, number of chronic diseases.

Table S10. Combined association of cooking fuel and kitchen ventilation with cognitive function.

	Cognitive score		Cognitive dysfunction	
	β (95% *CI*)	*P*	*OR* (95% *CI*)	*P*
**Model 1**				
Clean + good	Ref.		Ref.	
Clean + poor	-1.47 (-1.69, -1.25)	< 0.001	1.46 (1.32, 1.61)	< 0.001
Solid + good	-1.76 (-2.31, -1.21)	< 0.001	1.64 (1.30, 2.07)	< 0.001
Solid + poor	-3.19 (-3.50, -2.88)	< 0.001	2.08 (1.83, 2.37)	< 0.001
**Model 2**				
Clean + good	Ref.			
Clean + poor	-0.91 (-1.11, -0.71)	< 0.001	1.36 (1.23, 1.50)	< 0.001
Solid + good	-1.06 (-1.55, -0.57)	< 0.001	1.51 (1.20, 1.91)	0.001
Solid + poor	-1.86 (-2.14, -1.58)	< 0.001	1.82 (1.59, 2.08)	< 0.001
**Model 3**				
Clean + good	Ref.			
Clean + poor	-0.89 (-1.08, -0.69)	< 0.001	1.34 (1.22, 1.49)	< 0.001
Solid + good	-1.05 (-1.53, -0.56)	< 0.001	1.51 (1.19, 1.91)	0.001
Solid + poor	-1.80 (-2.08, -1.51)	< 0.001	1.78 (1.55, 2.04)	< 0.001

Short: cooking duration < 1.5 hours /day; Long: cooking duration ≥ 1.5 hours/day; Good: mechanical ventilation; Poor: natural ventilation.

Abbreviations: *β*, regression coefficient; *OR*, odds ratio; *CI*, confidence interval.

Model 1 was unadjusted.

Model 2 was adjusted for age, sex, marital status, education level, per capita monthly household income, smoking status, drinking status, physical activity level, high-fat diet, higher intake of fruits and vegetables, body mass index, and night sleep duration.

Model 3 was further adjusted for anxiety/depression, number of chronic diseases.

Table S11. Combined association of cooking fuel, daily cooking duration, and kitchen ventilation with cognitive function.

	Cognitive score		Cognitive dysfunction	
	β (95% *CI*)	*P*	*OR* (95% *CI*)	*P*
**Model 1**				
Clean + good + short	Ref.			
Clean + good + long	-0.62 (-0.93, -0.32)	< 0.001	1.04 (0.91, 1.20)	0.544
Clean + poor + short	-1.65 (-2.00, -1.30)	< 0.001	1.51 (1.29, 1.76)	< 0.001
Clean + poor + long	-1.97 (-2.28, -1.65)	< 0.001	1.49 (1.29, 1.71)	< 0.001
Solid + good + short	-1.95 (-2.85, -1.04)	< 0.001	1.50 (1.02, 2.20)	0.039
Solid + good + long	-2.25 (-2.96, -1.53)	< 0.001	1.80 (1.34, 2.43)	< 0.001
Solid + poor + short	-2.95 (-3.52, -2.38)	< 0.001	2.00 (1.57, 2.54)	< 0.001
Solid + poor + long	-3.79 (-4.19, -3.40)	< 0.001	2.19 (1.85, 2.59)	< 0.001
**Model 2**				
Clean + good + short	Ref.			
Clean + good + long	-0.33 (-0.61, -0.05)	0.021	1.04 (0.90, 1.21)	0.566
Clean + poor + short	-1.07 (-1.38, -0.76)	< 0.001	1.39 (1.19, 1.63)	< 0.001
Clean + poor + long	-1.13 (-1.41, -0.84)	< 0.001	1.39 (1.20, 1.61)	< 0.001
Solid + good + short	-0.97 (-1.76, -0.17)	0.017	1.38 (0.94, 2.04)	0.103
Solid + good + long	-1.42 (-2.05, -0.79)	< 0.001	1.66 (1.23, 2.24)	0.001
Solid + poor + short	-1.72 (-2.22, -1.21)	< 0.001	1.72 (1.34, 2.19)	< 0.001
Solid + poor + long	-2.17 (-2.53, -1.82)	< 0.001	1.92 (1.61, 2.29)	< 0.001
**Model 3**				
Clean + good + short	Ref.			
Clean + good + long	-0.34 (-0.62, -0.06)	0.016	1.05 (0.91, 1.22)	0.501
Clean + poor + short	-1.04 (-1.35, -0.73)	< 0.001	1.38 (1.18, 1.61)	< 0.001
Clean + poor + long	-1.11 (-1.40, -0.83)	< 0.001	1.39 (1.20, 1.60)	< 0.001
Solid + good + short	-0.94 (-1.73, -0.15)	0.02	1.37 (0.93, 2.02)	0.116
Solid + good + long	-1.43 (-2.06, -0.80)	< 0.001	1.67 (1.24, 2.27)	0.001
Solid + poor + short	-1.68 (-2.18, -1.17)	< 0.001	1.70 (1.33, 2.17)	< 0.001
Solid + poor + long	-2.12 (-2.48, -1.76)	< 0.001	1.88 (1.58, 2.25)	< 0.001

Short: cooking duration < 1.5 hours /day; Long: cooking duration ≥ 1.5 hours/day; Good: mechanical ventilation; Poor: natural ventilation.

Abbreviations: *β*, regression coefficient; *OR*, odds ratio; *CI*, confidence interval.

Model 1 was unadjusted.

Model 2 was adjusted for age, sex, marital status, education level, per capita monthly household income, smoking status, drinking status, physical activity level, high-fat diet, higher intake of fruits and vegetables, body mass index, and night sleep duration.

Model 3 was further adjusted for anxiety/depression, number of chronic diseases.

Table S12. The distribution of particulate matter concentration in the kitchen.

Particulate matter (ug/m^3)	N	Min	Max	Median	P_{25} – P_{75}	Mean	SD
Daily average kitchen $PM_{2.5}$	135	13.32	151.12	43.71	30.37 - 59.31	48.49	23.76
Daily average kitchen PM_{10}	135	15.22	169.25	51.21	35.29 - 69.79	56.91	28.11
Cooking-time-weighted kitchen $PM_{2.5}$	128	24.82	882.70	162.69	78.84 - 274.87	211.86	179.62
Cooking-time-weighted kitchen PM_{10}	128	27.99	1080.16	196.46	93.69 - 321.42	249.96	207.53

Abbreviations: N, number; Min, minimum value; Max, maximum value; P_{25}, 25th percentile; P_{75}, 75th percentile.; SD, standard deviation.

Table S13. Comparison of kitchen particulate matter concentrations among different cooking fuels, cooking durations, and ventilation types.

Cooking behavior	N	Daily average kitchen $PM_{2.5}$		Daily average kitchen PM_{10}	
		Median (P_{25}, P_{75})	*P*	Median (P_{25}, P_{75})	*P*
Cooking fuel	135				
Clean	111	41.45 (28.76, 53.31)	< 0.001	49.28 (33.31, 62.85)	< 0.001
Solid	24	66.02 (46.25, 85.04)		75.54 (56.20, 97.19)	
Cooking duration	129				
< 1.5 hours /day	37	28.86 (27.43, 37.60)	< 0.001	33.32 (31.07, 44.15)	< 0.001
⩾ 1.5 hours/day	92	51.73 (38.19, 65.40)		61.40 (43.26, 76.24)	
Kitchen ventilation	135				
Mechanical ventilation	74	39.58 (27.85, 53.54)	0.002	45.79 (32.16, 62.75)	0.002
Natural ventilation	61	48.40 (37.00, 66.40)		59.47 (42.22, 76.46)	

Abbreviations: N, number; P_{25}, 25th percentile; P_{75}, 75th percentile.

Table S14. Comparison of cooking-time-weighted kitchen particulate matter concentrations among different cooking fuels, cooking durations, and ventilation types.

Cooking behavior	N	Cooking-time-weighted kitchen $PM_{2.5}$		Cooking-time-weighted kitchen PM_{10}	
		Median (P_{25}, P_{75})	*P*	Median (P_{25}, P_{75})	*P*
Cooking fuel	128				
Clean	106	139.07 (70.74, 236.12)	< 0.001	168.90 (89.05, 281.43)	< 0.001
Solid	22	304.44 (221.33, 538.00)		361.99 (270.47, 630.14)	
Cooking duration	128				
< 1.5 hours /day	37	104.46 (60.23, 175.35)	< 0.001	121.38 (72.39, 230.57)	0.002
≥ 1.5 hours/day	91	195.02 (107.54, 303.60)		215.40 (131.64, 347.86)	
Kitchen ventilation	128				
Mechanical ventilation	70	122.49 (62.61, 205.20)	< 0.001	144.85 (78.23, 256.47)	< 0.001
Natural ventilation	58	213.66 (136.40, 314.25)		264.20 (156.20, 367.78)	

Abbreviations: N, number; P_{25}, 25th percentile; P_{75}, 75th percentile.

Table S15. The basic characteristics of the subjects included in the quantitative analysis.

Characteristics	Overall (N = 52)
Age (year), mean ± SD	68.62 ± 5.47
Gender, n (%)	
Man	13 (25.00)
Women	39 (75.00)
Marital status, n (%)	
Unmarried / Divorced / Separated / Widowed	0 (0.00)
Married/Cohabitation, n (%)	52 (100.00)
Education level, n (%)	
Elementary school below	16 (30.77)
Elementary school or above	36 (69.23)
Average monthly income (RMB), n (%)	
< 500	26 (50.00)
500~	16 (30.77)
1000~	10 (19.23)
Smoking status, n (%)	
Non-smoker	48 (92.31)
Current smoker	4 (7.69)
Drinking status, n (%)	
Non-drinker	52 (100.00)
Current drinker	0 (0.00)
Physical activity, n (%)	
Low	14 (26.92)
Moderate	24 (46.15)
High	14 (26.92)
High fat diet, n (%)	
No	41 (80.39)
Yes	10 (19.61)
High vegetable and fruit intake, n (%)	
No	13 (25.49)
Yes	38 (74.51)
BMI (kg/m^2), mean ± SD	23.96 ± 2.95
Night sleep duration (h/day), mean ± SD	7.72 ± 0.98
Anxiety/depression, n (%)	
No	49 (94.23)
Yes	3 (5.77)
Number of chronic diseases, n (%)	
0	9 (17.65)
1	18 (35.29)
2	15 (29.41)
3 -	9 (17.65)
MMSE (scores), mean ± SD	24.96 ± 2.92
Cooking fuel, n (%)	

Clean	32 (61.54)
Solid	20 (38.46)
Cooking duration, n (%)	
< 1.5 hours /day	16 (33.33)
≥ 1.5 hours/day	32 (66.67)
Kitchen ventilation, n (%)	
Mechanical ventilation	19 (36.54)
Natural ventilation	33 (63.46)
Daily average kitchen $PM_{2.5}$ ($\mu g/m^3$), median (P_{25}, P_{75})	45.62 (33.45, 66.48)
Daily average kitchen PM_{10} ($\mu g/m^3$), median (P_{25}, P_{75})	55.44 (38.37, 77.23)
Cooking-time-weighted kitchen $PM_{2.5}$ ($\mu g/m^3$), median (P_{25}, P_{75})	231.43 (125.99, 359.82)
Cooking-time-weighted kitchen PM_{10} ($\mu g/m^3$), median (P_{25}, P_{75})	272.52 (138.48, 408.37)

Abbreviations: SD, standard deviation; P_{25}, 25th percentile; P_{75}, 75th percentile; BMI, body mass index.

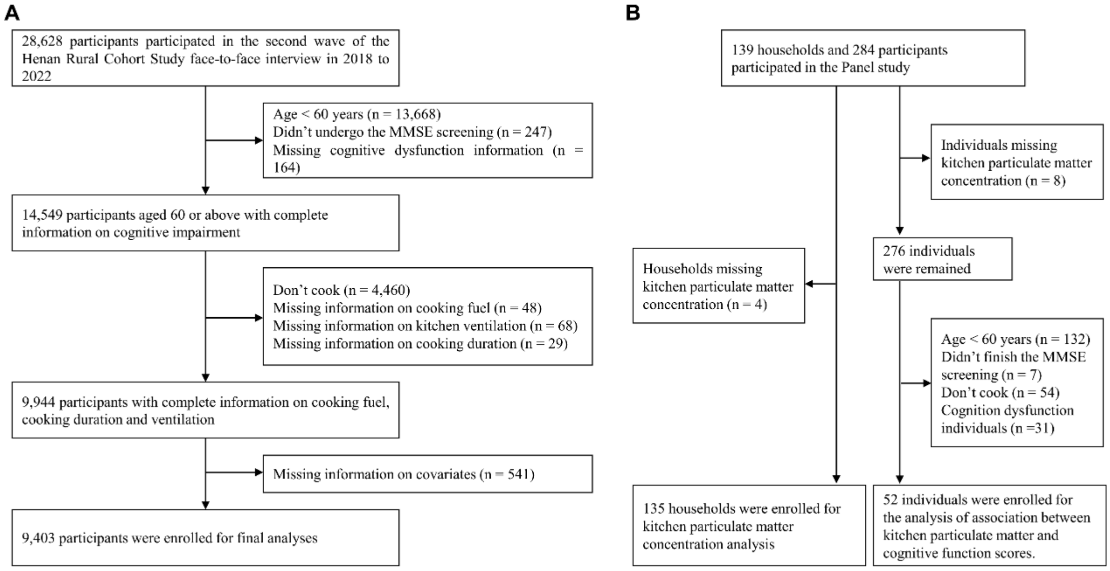

Figure S1. Flow chart of study participants (A: Flow chart of qualitative study participants; B: Flow chart of quantitative study participants).

Abbreviations: MMSE, Mini-Mental State Examination.

Subgroup	N	Cognitive score β (95% CI)	Cognitive score P for interaction	Cognitive dysfunction OR (95% CI)	Cognitive dysfunction P for interaction
**Age**					
< 70	5,740	-0.98 (-1.29, -0.68)	-	1.41 (1.21, 1.65)	-
70~	3,663	-1.53 (-1.91, -1.14)	0.019	1.58 (1.33, 1.88)	0.153
**Sex**					
Man	2,205	-1.24 (-1.73, -0.76)	-	1.48 (1.16, 1.90)	-
Women	7,198	-1.15 (-1.43, -0.88)	0.789	1.47 (1.29, 1.67)	0.869
**Marital status**					
Unmarried/divorced/widowed	2,129	-1.39 (-1.92, -0.87)	-	1.32 (1.04, 1.68)	-
Married/cohabitation	7,274	-1.10 (-1.37, -0.84)	0.29	1.51 (1.33, 1.72)	0.546
**Educational level**					
Illiteracy	3,279	-1.36 (-1.77, -0.95)	-	1.66 (1.39, 1.97)	-
Elementary school or above	6,124	-1.01 (-1.30, -0.71)	0.071	1.31 (1.12, 1.52)	0.011
**Average monthly income**					
< 500	3,961	-1.33 (-1.68, -0.99)	-	1.54 (1.32, 1.80)	-
500~	2,545	-0.89 (-1.36, -0.42)	0.112	1.24 (0.98, 1.56)	0.137
1000~	2,897	-1.14 (-1.63, -0.64)	0.589	1.54 (1.19, 1.98)	0.827
**Smoking status**					
Never/former smoker	8,548	-1.19 (-1.44, -0.94)	-	1.46 (1.30, 1.65)	-
Current smoker	855	-1.14 (-1.92, -0.36)	0.823	1.62 (1.07, 2.45)	0.867
**Drinking status**					
Never/former drinker	8,698	-1.15 (-1.43, -0.88)	-	1.47 (1.29, 1.67)	-
Current drinker	705	-1.48 (-2.36, -0.59)	0.475	1.33 (0.82, 2.14)	0.553
**Physical activity**					
Low	3,552	-1.08 (-1.47, -0.69)	-	1.44 (1.20, 1.72)	-
Moderate	3,975	-1.43 (-1.80, -1.06)	0.234	1.51 (1.25, 1.81)	0.716
High	1,876	-0.82 (-1.35, -0.29)	0.233	1.41 (1.09, 1.81)	0.699
**High fat diet**					
No	8,049	-1.27 (-1.52, -1.01)	-	1.50 (1.33, 1.70)	-
Yes	1,354	-0.55 (-1.25, 0.16)	0.063	1.19 (0.82, 1.73)	0.232
**More vegetable and fruit intake**					
No	5,294	-1.41 (-1.73, -1.09)	-	1.47 (1.27, 1.71)	-
Yes	4,109	-0.84 (-1.20, -0.48)	0.009	1.45 (1.21, 1.74)	0.544
**BMI**					
< 24.0	4,223	-1.23 (-1.56, -0.89)	-	1.51 (1.28, 1.77)	-
24.0~	5,180	-1.14 (-1.48, -0.80)	0.522	1.44 (1.22, 1.69)	0.755
**Night sleep duration**					
< 6	2,691	-0.89 (-1.32, -0.47)	0.084	1.20 (0.96, 1.50)	0.011
7~	3,092	-1.30 (-1.73, -0.87)	-	1.65 (1.34, 2.02)	-
8~	2,360	-1.01 (-1.49, -0.53)	0.298	1.44 (1.14, 1.80)	0.222
9~	1,260	-1.62 (-2.28, -0.96)	0.244	1.69 (1.27, 2.24)	0.764
**Anxiety/depression**					
No	8,844	-1.12 (-1.37, -0.87)	-	1.45 (1.28, 1.63)	-
Yes	559	-1.79 (-2.62, -0.96)	0.11	1.68 (1.14, 2.46)	0.506
**Number of chronic diseases**					
0	2,722	-1.30 (-1.73, -0.87)	-	1.54 (1.25, 1.89)	-
1	3,301	-1.02 (-1.43, -0.61)	0.302	1.41 (1.16, 1.72)	0.583
2	2,157	-1.38 (-1.88, -0.88)	0.858	1.41 (1.11, 1.80)	0.559
3 ~	1,223	-0.84 (-1.53, -0.15)	0.123	1.49 (1.08, 2.03)	0.54

Figure S2. Stratification analyses of the association between cooking fuel and cognitive function

Abbreviations: N, number; *β*, regression coefficient; *OR*, odds ratio; *CI*, confidence interval; BMI, body mass index.

The model was adjusted for age, sex, marital status, education level, per capita monthly household income, smoking status, drinking status, physical activity level, high-fat diet, higher intake of fruits and vegetables, BMI, night sleep duration, anxiety/depression, and number of chronic diseases, in addition to the stratification factors.

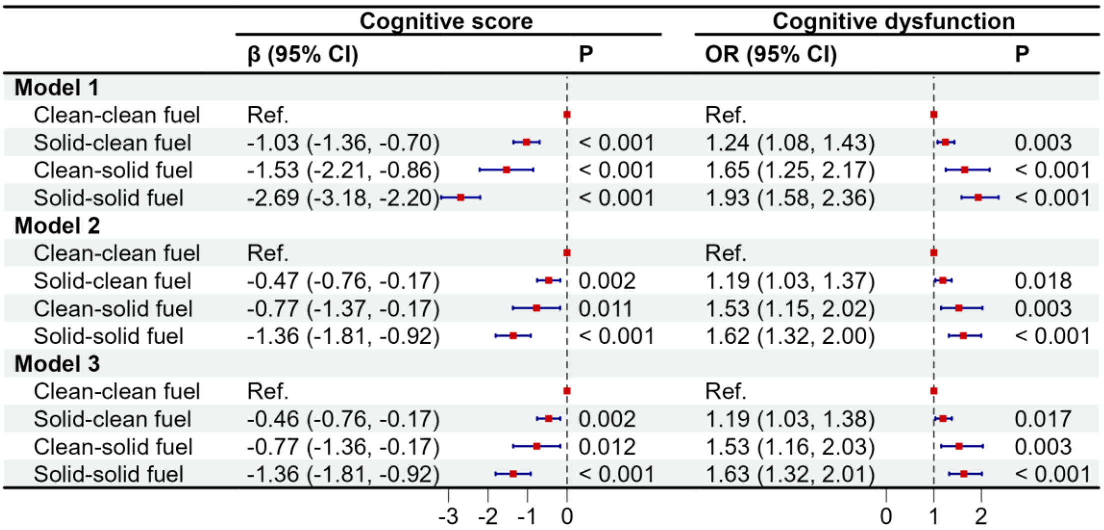

	Cognitive score		Cognitive dysfunction	
	β (95% CI)	P	OR (95% CI)	P
**Model 1**				
Clean-clean fuel	Ref.		Ref.	
Solid-clean fuel	-1.03 (-1.36, -0.70)	< 0.001	1.24 (1.08, 1.43)	0.003
Clean-solid fuel	-1.53 (-2.21, -0.86)	< 0.001	1.65 (1.25, 2.17)	< 0.001
Solid-solid fuel	-2.69 (-3.18, -2.20)	< 0.001	1.93 (1.58, 2.36)	< 0.001
**Model 2**				
Clean-clean fuel	Ref.		Ref.	
Solid-clean fuel	-0.47 (-0.76, -0.17)	0.002	1.19 (1.03, 1.37)	0.018
Clean-solid fuel	-0.77 (-1.37, -0.17)	0.011	1.53 (1.15, 2.02)	0.003
Solid-solid fuel	-1.36 (-1.81, -0.92)	< 0.001	1.62 (1.32, 2.00)	< 0.001
**Model 3**				
Clean-clean fuel	Ref.		Ref.	
Solid-clean fuel	-0.46 (-0.76, -0.17)	0.002	1.19 (1.03, 1.38)	0.017
Clean-solid fuel	-0.77 (-1.36, -0.17)	0.012	1.53 (1.16, 2.03)	0.003
Solid-solid fuel	-1.36 (-1.81, -0.92)	< 0.001	1.63 (1.32, 2.01)	< 0.001

Figure S3. Association of cooking fuel transition with cognitive function.

Abbreviation: *β*, regression coefficient; *OR*, odds ratio; *CI*, confidence interval.

Model 1 was unadjusted.

Model 2 was adjusted for age, sex, marital status, education level, per capita monthly household income, smoking status, drinking status, physical activity level, high-fat diet, higher intake of fruits and vegetables, body mass index, and night sleep duration.

Model 3 was further adjusted for anxiety/depression, number of chronic diseases.

	Cognitive score		Cognitive dysfunction	
	β (95% CI)	P	OR (95% CI)	P
**Model 1**				
Clean fuel ≤ 10 years	Ref.		Ref.	
Clean fuel > 10 years	-0.13 (-0.39, 0.13)	0.327	1.09 (0.98, 1.22)	0.13
Solid fuel ≤ 10 years	-2.16 (-2.70, -1.63)	< 0.001	1.60 (1.29, 2.00)	< 0.001
Solid fuel > 10 years	-2.24 (-2.55, -1.93)	< 0.001	1.68 (1.48, 1.90)	< 0.001
**Model 2**				
Clean fuel ≤ 10 years	Ref.		Ref.	
Clean fuel > 10 years	-0.28 (-0.51, -0.05)	0.017	1.12 (1.00, 1.26)	0.052
Solid fuel ≤ 10 years	-1.35 (-1.82, -0.88)	< 0.001	1.47 (1.18, 1.84)	0.001
Solid fuel > 10 years	-1.27 (-1.54, -1.00)	< 0.001	1.52 (1.34, 1.74)	< 0.001
**Model 3**				
Clean fuel ≤ 10 years	Ref.		Ref.	
Clean fuel > 10 years	-0.28 (-0.51, -0.05)	0.018	1.12 (1.00, 1.25)	0.059
Solid fuel ≤ 10 years	-1.29 (-1.76, -0.82)	< 0.001	1.44 (1.15, 1.80)	0.001
Solid fuel > 10 years	-1.23 (-1.50, -0.96)	< 0.001	1.50 (1.32, 1.71)	< 0.001

Figure S4. Association of cooking fuel usage time with cognitive function.

Abbreviation: *β*, regression coefficient; *OR*, odds ratio; *CI*, confidence interval.

Model 1 was unadjusted.

Model 2 was adjusted for age, sex, marital status, education level, per capita monthly household income, smoking status, drinking status, physical activity level, high-fat diet, higher intake of fruits and vegetables, body mass index, and night sleep duration.

Model 3 was further adjusted for anxiety/depression, number of chronic diseases.

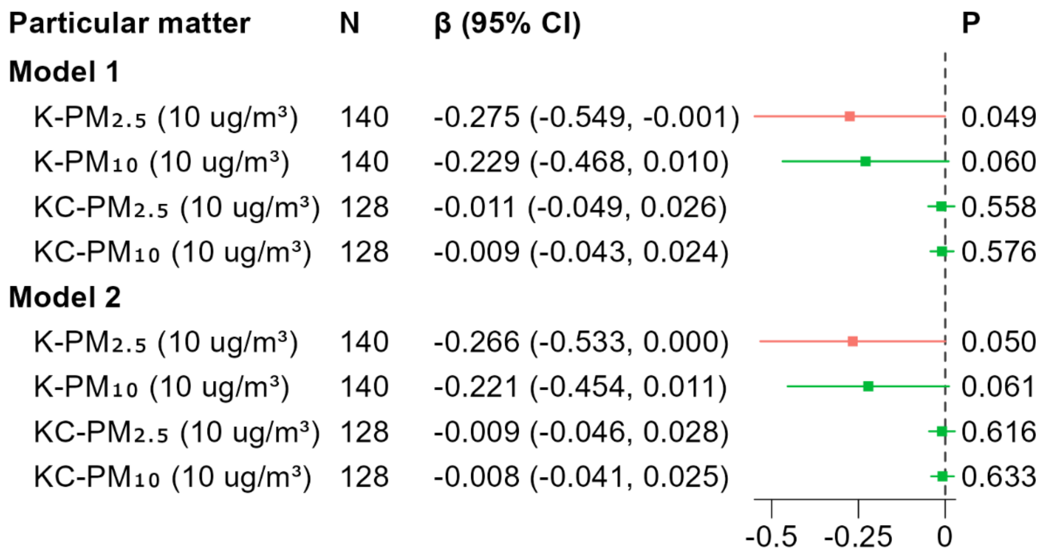

Figure S5. Association of kitchen particulate matter and cognitive function scores.

Abbreviations: K-$PM_{2.5}$, Daily average kitchen $PM_{2.5}$; K-PM_{10}, Daily average kitchen PM_{10}; KC-$PM_{2.5}$, Cooking-time-weighted kitchen $PM_{2.5}$; KC-PM_{10}, Cooking-time-weighted kitchen PM_{10}; β, regression coefficient; *CI*, confidence interval.

Model 1 was unadjusted.

Model 2 was adjusted for age and sex.
